# Validation of preoperative *BRAF* V600E testing by ThyroSCAN PanelChip in thyroid nodules

**DOI:** 10.1530/EC-24-0718

**Published:** 2025-05-16

**Authors:** Po-Sheng Lee, Jui-Yu Chen, Chia-Chin Lee, Li-Hsin Pan, Jen-Fan Hang, Po-Chung Kuo, Shan-Fan Yao, Chii-Min Hwu, Chin-Sung Kuo

**Affiliations:** ^1^ Section of Endocrinology and Metabolism, Department of Medicine, Taipei Veterans General Hospital, Taipei, Taiwan; ^2^ School of Medicine, National Yang Ming Chiao Tung University, Taipei, Taiwan; ^3^ Division of General Surgery, Department of Surgery, Taipei Veterans General Hospital, Taipei, Taiwan; ^4^ Institute of Biology and Anatomy, National Defense Medical Center, Taipei, Taiwan; ^5^ Section of Endocrinology and Metabolism, Department of Medicine, Taipei City Hospital Zhongxing Branch, Taipei, Taiwan; ^6^ Department of Pathology and Laboratory Medicine, Taipei Veterans General Hospital, Taipei, Taiwan; ^7^ Department of Pathology, School of Medicine, National Yang Ming Chiao Tung University, Taipei, Taiwan; ^8^ Institute of Clinical Medicine, National Yang Ming Chiao Tung University, Taipei, Taiwan; ^9^ Department of Nuclear Medicine, Taipei Veterans General Hospital, Taipei, Taiwan

**Keywords:** *BRAF* V600E mutation, molecular genetic testing, preoperative diagnostics, thyroid nodules

## Abstract

**Objectives:**

The high cost of preoperative molecular testing remains a significant barrier to their widespread clinical use. This prospective study aims to investigate the clinical applicability of the ThyroSCAN PanelChip, a qPCR-based method, for preoperative *BRAF* V600E testing in thyroid nodules.

**Materials and methods:**

Adult patients undergoing fine-needle aspiration cytology in outpatient settings between April 2023 and June 2024 were enrolled, and molecular testing was performed on aspiration samples. For patients who proceeded to thyroidectomy, postoperative histopathological findings were compared with preoperative cytology and molecular results. Immunohistochemical VE1 staining on pathology specimens served as the gold standard to validate accuracy. A best-estimate approach was also employed to expand the evaluation, including presumed *BRAF* V600E-negative cases, such as *RAS*-positive specimens and benign lesions.

**Results:**

Among 73 patients who underwent thyroidectomy and were included in the analysis, preoperative molecular testing identified *BRAF* mutations in 22 patients, detected the wild-type gene in 39 and classified 12 as inaccessible due to insufficient DNA extraction. For the 38 patients with both preoperative genetic results and VE1 staining, performance metrics were: positive predictive value = 95.5%, negative predictive value = 75.0%, accuracy = 86.8%, sensitivity = 84.0% and specificity = 92.3%. Under the best-estimate approach, metrics improved to positive predictive value = 95.5%, negative predictive value = 89.5%, accuracy = 91.7%, sensitivity = 84.0% and specificity = 97.1%.

**Conclusion:**

These findings demonstrate that ThyroSCAN PanelChip effectively identifies *BRAF* V600E mutations in thyroid nodules using residual thyrocytes from fine-needle aspiration samples.

## Introduction

Thyroid nodules are a prevalent clinical finding, affecting 19–68% of the population (1). Although most nodules are benign and asymptomatic, about 5–15% may be malignant, underscoring the need for accurate risk assessment to ensure timely and appropriate treatment (1, 2, 3). Fine-needle aspiration (FNA) cytology serves as the primary diagnostic tool for evaluating thyroid nodules; however, managing cases with indeterminate cytology remains a significant challenge (4, 5).

The *BRAF* V600E mutation, known for its high specificity in malignancy, is detected in approximately 61.7% of papillary thyroid carcinoma (PTC), 1.7% of follicular thyroid carcinoma (FTC), 19–45% of anaplastic thyroid carcinoma and 19–33% of poorly differentiated thyroid carcinoma (PDTC) (6). In Taiwan, a notably high prevalence of *BRAF* V600E mutation in PTC (86%) has been reported, with no significant correlation to disease aggressiveness (7, 8). This mutation has become a critical marker, enhancing both diagnostic and prognostic evaluations, especially in cases of indeterminate thyroid nodules (9, 10, 11). However, the high cost of preoperative molecular testing methods, such as ThyroSeq v3 or Afirma, remains a significant barrier to their widespread clinical use outside of North America (12).

Quantitative polymerase chain reaction (qPCR) has emerged as a well-established and cost-effective method for mutation testing (13). The ThyroSCAN single nucleotide variant (SNV) PanelChip is a commercially available kit utilizing the qPCR technique to detect SNVs, including the *BRAF* V600E mutation. However, insufficient DNA extraction from residual thyrocytes in FNA syringes and the technical challenges of performing FNA on small nodules are significant factors that may affect the accuracy of molecular testing. To address these issues, we conducted a prospective study to validate the preoperative detection of *BRAF* V600E mutations in thyroid nodules using the ThyroSCAN PanelChip.

## Materials and methods

### Study design and patient selection

This prospective study was conducted at Taipei Veterans General Hospital, Taiwan, from April 2023 to June 2024. It enrolled adult participants (≥20 years) with thyroid nodules who underwent thyroid ultrasonography and FNA cytology in outpatient settings, providing written informed consent. Exclusion criteria included pregnancy or age under 20. Residual FNA samples were used for molecular testing. The study was approved by the Institutional Review Board of Taipei Veterans General Hospital (IRB No.: 2023-01-020CC) and conducted in accordance with the Declaration of Helsinki.

### Fine-needle aspiration

FNA was performed by endocrinologists using a high-resolution 14 MHz linear-array transducer, covered with a sterile sheath and ultrasound gel. A 22-gauge needle was employed for specimen collection, with patients positioned in a supine posture. After a preliminary ultrasound to determine the nodule location, three aspirations were performed under real-time ultrasound guidance, and cytology slides were prepared using a blood smear technique followed by Liu’s stain. Rapid on-site evaluation was conducted to ensure cellular adequacy, with an additional fourth pass performed if necessary.

### Molecular testing

All FNA specimens, regardless of their classification under the Bethesda system (TBS), underwent molecular genetic analysis. Residual FNA samples were preserved in CytoRich Red Collection Fluid (Thermo Fisher Scientific, USA) after cytological smear preparation and transported at 4°C. Sample pellets were obtained by centrifugation at 2,000 ***g*** for 5 min, followed by the removal of CytoRich Red. The pellets were washed with PBS buffer, centrifuged again at 2,000 ***g*** for 5 min and PBS was removed. Total nucleic acid isolation was performed using the RecoverAll™ Multi-Sample RNA/DNA Isolation Workflow (Invitrogen™, Thermo Fisher Scientific, USA), following the manufacturer’s protocol. The isolated DNA was quantified using the Qubit™ dsDNA HS Assay Kit (Invitrogen™, Thermo Fisher Scientific, USA). For *BRAF* V600E detection, DNA preamplification was conducted in a mixture (S1) containing 35 ng DNA and the ThyroSCAN Cancer Detection Kit (QuarkBio, Taiwan). The preamplified S1 was diluted 20-fold before proceeding to qPCR. A 15 μL aliquot of the diluted S1 was added to the qPCR mixture and loaded onto the ThyroSCAN SNV PanelChip, which is designed for the mutation detection of *BRAF* V600E, *HRAS* Q61K, *HRAS* Q61R, *NRAS* Q61K and *NRAS* Q61R. The competitive allele-specific TaqMan PCR experiments were carried out using the ThyroSCAN APP and the SNV PanelChip on the Q Station™ 1000 platform.

### Immunohistochemical validation

For patients who underwent thyroidectomy, postoperative histopathological findings were compared with preoperative FNA cytology and molecular test results, according to the fifth edition of the WHO Classification of Endocrine and Neuroendocrine Tumors, released in 2022 (14). Immunohistochemistry (IHC) on formalin-fixed, paraffin-embedded (FFPE) thyroidectomy specimens served as the definitive standard for validating the accuracy of molecular analyses, utilizing staining for *BRAF* (clone: VE1, Ventana, USA). In our routine pathological practice, the VE1 staining were performed at discretion of the on-duty pathologist, typically in cases suspected of or confirmed as PTC based on histopathological morphology. To ensure an unbiased histopathological evaluation, pathologists were unaware of the preoperative molecular testing results. In certain conditions, e.g., follicular-patterned lesions but lacked definitive PTC characteristics, the pathologist also performed IHC staining for *RAS* Q61R (clone: RBT-NRAS, Bio SB, USA).

### Performance metrics and best-estimate approach

The diagnostic efficacy of the ThyroSCAN panel in detecting *BRAF* V600E was assessed by calculating performance metrics comprising accuracy, sensitivity, specificity, positive predictive value (PPV) and negative predictive value (NPV), in comparison with results of IHC VE1 staining of pathology. In addition to cases with definitive VE1 results, a best-estimate approach was employed to broaden the evaluation by including cases reasonably inferred to be *BRAF* V600E negative, such as *RAS*-positive specimens, reflecting the mutually exclusive nature between *RAS* and *BRAF* mutations and benign thyroid lesions.

### Statistics

All values of performance metrics are reported with corresponding 95% confidence interval (CI), calculated using the Wilson score method. Genetic characteristics, including the prevalence of insufficient genetic material and *BRAF/RAS* mutations, and pathological differences across TBS categories, were compared using the Fisher’s exact test. Statistical analyses were performed using the IBM SPSS Statistics (Version 26.0, IBM Corp., USA), with a two-sided significance threshold of *P* < 0.05.

## Results

**Figure 1:**
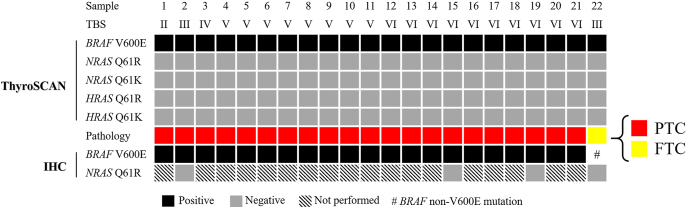
Preoperative *BRAF* positivity detected by ThyroSCAN, with corresponding IHC outcomes from FFPE samples.

**Figure 2:**
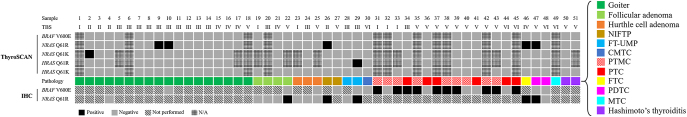
Preoperative *BRAF*-negative results by ThyroSCAN, alongside IHC findings from FFPE thyroid tissue.

Of the 158 enrolled patients with thyroid nodules who underwent FNA, 47 of them were categorized as indeterminate, TBS III 39 (24.7%), TBS IV 8 (5.1%) and about one fourth as TBS V 26 (16.5%) or VI 16 (10.1%) (Supplemental Table 1 (see section on Supplementary materials given at the end of the article)). The 73 out of 158 who proceeded to thyroidectomy were included in the analysis. Most of them were classified as suspicious malignancy or malignancy: TBS I 6 (8.2%), TBS II 5 (6.8%), TBS III 17 (23.3%), TBS IV 6 (8.2%), TBS V 24 (32.9%) and TBS VI 15 (20.5%) (see Table 1). Preoperative ThyroSCAN molecular analysis detected *BRAF* mutations in 22 patients, identified the wild-type gene in 39 patients and classified 12 cases as unavailable (N/A) due to insufficient DNA extraction concentrations, which did not meet the minimum input requirement for the PanelChip test despite standard preamplification procedures (Figs 1 and 2). The preoperative molecular testing results and final pathology findings for the patients undergoing thyroidectomy are summarized in Table 1, with higher TBS categories (TBS V and VI) exhibiting greater proportions of *BRAF* mutations, PTC diagnoses and malignancy rates.

**Table 1:** Preoperative molecular testing and final pathology results of patients undergoing thyroidectomy across TBS categories.

	TBS I	TBS II	TBS III	TBS IV	TBS V	TBS VI	*P* value
Total patients (*n*/%)	6 (8.2%)	5 (6.8%)	17 (23.3%)	6 (8.2%)	24 (32.9%)	15 (20.5%)	-
Insufficient genetic material^†^ (*n*/%)	3 (50.0%)	0 (0.0%)	2 (11.7%)	0 (0.0%)	5 (20.8%)	2 (13.3%)	0.262
Mutation non-detected^‡^ (*n*/%)	3 (50.0%)	2 (40.0%)	10 (58.8%)	4 (66.7%)	10 (41.7%)	3 (20.0%)	0.255
*BRAF* V600E mutation (*n*/%)	0 (0.0%)	1 (20.0%)	2 (11.7%)	1 (16.7%)	8 (33.3%)	10 (66.7%)	0.008*
*RAS* mutation (*n*/%)							0.109
*NRAS* (*n*/%)	0 (0.0%)	2 (40.0%)	2 (11.7%)	1 (16.7%)	1 (4.2%)	0 (0.0%)	
*HRAS* (*n*/%)	0 (0.0%)	0 (0.0%)	1 (5.9%)	0 (0.0%)	0 (0.0%)	0 (0.0%)	
Pathology (*n*/%)							<0.001*
Goiter	1 (16.7%)	3 (60.0%)	10 (58.8%)	3 (50.0%)	1 (4.2%)	0 (0.0%)	
FA	1 (16.7%)	0 (0.0%)	1 (5.9%)	1 (16.7%)	1 (4.2%)	0 (0.0%)	
HCA	1 (16.7%)	0 (0.0%)	1 (5.9%)	0 (0.0%)	1 (4.2%)	0 (0.0%)	
NIFTP	0 (0.0%)	1 (20.0%)	0 (0.0%)	0 (0.0%)	1 (4.2%)	0 (0.0%)	
FT-UMP	0 (0.0%)	0 (0.0%)	2 (11.8%)	0 (0.0%)	0 (0.0%)	0 (0.0%)	
HT	0 (0.0%)	0 (0.0%)	0 (0.0%)	0 (0.0%)	2 (8.3%)	0 (0.0%)	
PTC	3 (50.0%)	1 (20.0%)	2 (11.8%)	1 (16.7%)	17 (70.8%)	12 (80.0%)	
FTC	0 (0.0%)	0 (0.0%)	1 (5.9%)	1 (16.7%)	0 (0.0%)	0 (0.0%)	
PDTC	0 (0.0%)	0 (0.0%)	0 (0.0%)	0 (0.0%)	1 (4.2%)	1 (6.7%)	
CMTC	0 (0.0%)	0 (0.0%)	0 (0.0%)	0 (0.0%)	0 (0.0%)	1 (6.7%)	
MTC	0 (0.0%)	0 (0.0%)	0 (0.0%)	0 (0.0%)	0 (0.0%)	1 (6.7%)	
ROM (*n*/%)	3 (50.0%)	1 (20.0%)	3 (17.6%)	2 (33.3%)	18 (66.7%)	15 (100.0%)	<0.001*

* These *P* values indicate statistical significance.

^†^ Prevent ThyroSCAN™ from producing meaningful data.

^‡^ Wild-type gene observed in the specimen; TBS, the Bethesda system, ROM, risk of malignancy; FA, follicular adenoma; HCA, Hurthle cell adenoma; NIFTP, noninvasive follicular neoplasm with papillary-like nuclear features; FT-UMP, follicular tumor of uncertain malignant potential; HT, Hashimoto’s thyroiditis; PTC, papillary thyroid carcinoma; FTC, follicular thyroid carcinoma; PDTC, poorly differentiated thyroid carcinoma; CMTC, cribriform morular thyroid carcinoma; MTC, medullary thyroid cancer.

Among the 73 cases that underwent thyroidectomy, 42 were diagnosed as malignant, yielding a cancer prevalence of 57.5%. Of these, 36 cases were confirmed as PTC (Table 1), including nine classified as papillary thyroid microcarcinoma (PTMC) (<1 cm). Among the 36 PTC cases, 31 (86.1%) were VE1-positive (Figs 1 and 2). In addition, 15 PTC cases (including nine PTMC) did not exhibit a preoperative *BRAF* V600E mutation (Fig. 2).

For the nine PTMC cases, five with N/A preoperative *BRAF* results showed 80.0% VE1 positivity (4/5), while four with negative preoperative *BRAF* results showed 75.0% VE1 positivity (3/4). Among the remaining six PTC cases (excluding PTMC), two with N/A preoperative *BRAF* results demonstrated 100.0% VE1 positivity, whereas four cases with negative preoperative *BRAF* results showed 25.0% false negatives, with one of the four being VE1-positive (Fig. 2). The N/A rate for preoperative *BRAF* testing in all 73 analyzed cases was 16.4% (12/73), 13.5% (5/37) in non-PTC, 7.4% (2/27) in all PTC cases (excluding PTMC) and 55.6% (5/9) in PTMC cases (Fig. 2).

Among the 39 patients with no preoperative *BRAF* V600E mutation, VE1 staining was performed in 16 cases: 12 were VE1-negative and four were VE1-positive (Fig. 2). For the 38 patients with both preoperative *BRAF* mutation analysis and VE1 IHC staining results, the performance metrics of the qPCR-based ThyroSCAN SNV PanelChip for detecting *BRAF* V600E were as follows: PPV = 21/22 (0.955, 95% CI: 0.782–0.992), NPV = 12/16 (0.750, 95% CI: 0.505–0.898), accuracy = 33/38 (0.868, 95% CI: 0.727–0.942), sensitivity = 21/25 (0.840, 95% CI: 0.653–0.936) and specificity = 12/13 (0.923, 95% CI: 0.667–0.986) (Tables 2 and 3).

**Table 2:** Performance of ThyroSCAN for *BRAF* V600E detection validated by IHC VE1 and best-estimate approach.

ThyroSCAN™	VE1 positive	VE1 negative	Best-estimate approach as *BRAF* V600E negative
	*n*	*n*	*n*
*BRAF* V600E (+)	21	1	1
*BRAF* V600E (−)	4	12	34

**Table 3:** Performance metrics of ThyroSCAN™ for *BRAF* V600E evaluation.

	Sensitivity	Specificity	PPV	NPV	Accuracy
Performance with definite VE1 (*n* = 38)	0.840 (0.653–0.936)	0.923 (0.667–0.986)	0.955 (0.782–0.992)	0.750 (0.505–0.898)	0.868 (0.727–0.942)
Best-estimate approach (*n* = 60)	0.840 (0.653–0.936)	0.971 (0.855–0.995)	0.955 (0.782–0.992)	0.895 (0.759–0.958)	0.917 (0.819–0.964)

PPV, positive predictive value; NPV, negative predictive value.

Performance metrics were calculated using the Wilson score method, presented as estimates with 95% confidence intervals.

We further evaluated the best-estimate performance by incorporating an additional 20 cases without VE1 data, which were confirmed as benign pathology and thus classified as *BRAF* V600E-negative. In addition, two cases with positive IHC *RAS* staining, which were considered *BRAF* V600E-negative due to mutual exclusivity, were included. Under these expanded conditions, the preoperative *BRAF* V600E performance metrics using ThyroSCAN SNV PanelChip were as follows: PPV = 21/22 (0.955, 95% CI: 0.782–0.992), NPV = 34/38 (0.895, 95% CI: 0.759–0.958), accuracy = 55/60 (0.917, 95% CI: 0.819–0.964), sensitivity = 21/25 (0.840, 95% CI: 0.653–0.936) and specificity = 34/35 (0.971, 95% CI: 0.855–0.995) (Tables 2 and 3).

Of the 22 *BRAF* V600E-positive cases, 21 were confirmed as PTC, all of which were positive by VE1 IHC staining, and one case was identified as VE1-negative FTC (Fig. 1). The discrepancy between preoperative qPCR results for *BRAF* V600E and VE1 IHC findings prompted further investigation with Sanger sequencing, revealing a rare *BRAF* mutation (V600_K601insNTV) in the FTC case, as previously reported (15).

In the TBS I group, half of the cases (50.0%) had insufficient genetic material for analysis, while the remaining 50.0% showed no detectable mutations. In contrast, no cases in the TBS II group had insufficient genetic material. Within TBS I, three cases of PTC were identified, whereas TBS II included one case of *BRAF*-positive PTC. The ROM was calculated at 50.0% for TBS I and 20.0% for TBS II. *BRAF* positivity and ROM increased progressively across TBS categories from TBS III to TBS VI, with malignancy rate of 100.0% and *BRAF* prevalence of 66.7% in the TBS VI group. Most malignancies were PTC, accounting approximately for 70–80% of cases in the two highest TBS categories. Conversely, the prevalence of benign thyroid lesions, such as goiter, decreased from 58.8% in TBS III to 0.0% in TBS VI, consistent with the TBS grading pattern.

*RAS* mutations were detected in seven patients, with three cases of goiter, one noninvasive follicular thyroid neoplasm with papillary-like nuclear features (NIFTP), one follicular tumor of uncertain malignant potential (FT-UMP), one FTC and one PDTC. IHC was not performed for the goiter cases due to their clear benign appearance under histopathology; however, *RAS* mutations in the remaining cases were confirmed via IHC on FFPE tissue (Fig. 2).

## Discussion

This study represents the first preoperative investigation of *BRAF* V600E mutations in thyroid nodules conducted in Taiwan. Our results demonstrated that the ThyroSCAN PanelChip effectively identified *BRAF* V600E mutations with the following performance metrics: PPV = 21/22 (95.5%), NPV = 12/16 (75.0%), accuracy = 33/38 (86.8%), sensitivity = 21/25 (84.0%) and specificity = 12/13 (92.3%). Using the best-estimate approach, the performance metrics improved to PPV = 21/22 (95.5%), NPV = 34/38 (89.5%), accuracy = 55/60 (91.7%), sensitivity = 21/25 (84.0%) and specificity = 34/35 (97.1%). The participants with PTMC were with high N/A rate 55.6% (5/9) of preoperative *BRAF* V600E testing. This study not only enhances the understanding of preoperative molecular testing for thyroid nodules but also contributes to the report of the first case of FTC with a novel *BRAF* (V600_K601insNTV) mutation presenting as preoperative *BRAF* V600E false-positive (15).

In this study, the enrolled patients had a high proportion of indeterminate thyroid nodules in addition to approximately one-fourth classified as TBS V or VI. A nationwide survey in Taiwan indicated that most FNA cytology results were categorized as TBS I and II (16). Similarly, previous FNA cytology reports from the Taipei Veterans General Hospital showed most classified as TBS I (8%) and II (81%) (17). This study was conducted at a tertiary medical center, and the enrolled patients demonstrated a significantly higher malignancy risk in FNA results compared to prior hospital reports (17). Selection bias may have influenced the enrollment process, as patients with prominent benign lesions were likely excluded based on clinical judgment, while cases with higher clinical suspicion were encouraged to participate. However, this potential selection bias is unlikely to have affected the validity of the study’s findings.

The qPCR-based ThyroSCAN panel exhibited performance metrics comparable to other *BRAF* detection methods, achieving a sensitivity of 84.0% and a specificity of 92.3% in this study. For instance, Kwak *et al.* reported that dual priming oligonucleotide (DPO)-based multiplex PCR, direct DNA sequencing and PCR-restriction fragment length polymorphism (RFLP), yielded sensitivities of 79.1, 69.1 and 67.3%, respectively, all with a specificity of 100.0% (18). Similarly, droplet digital PCR (ddPCR) *BRAF* V600E testing in frozen FNA samples showed a sensitivity of 91.3% and specificity of 100.0%, while amplification refractory mutation system (ARMS)-PCR achieved a sensitivity of 83.1% and specificity of 100.0% (19).

A recent study by Fu *et al.* utilized locked nucleic acid probe-based ddPCR to analyze the variant allele frequency (VAF) of *BRAF* V600E and *TERT* promoter mutations in 217 residual thyroid FNA specimens, demonstrating high sensitivity 93.8% and strong concordance with surgical tissue results (20). The ddPCR method allows for absolute quantification, making it possible to detect low-VAF mutations (<1%). This capability supports early diagnosis and helps exclude low-VAF cases, thereby identifying patients at medium to high risk of recurrence (20). In contrast, this study used a simple and efficient qPCR chip to assess whether FNA specimens from thyroid nodules contain the *BRAF* V600E mutation, along with four other gene mutations. Unlike ddPCR methods, the ThyroSCAN PanelChip does not perform VAF analysis but still provides valuable information for physicians when formulating patient treatment plans.

Priced at approximately $200–300 for research, ThyroSCAN based on qPCR method is cost-effective for routine analyses and large-scale studies. Nonetheless, insufficient DNA extraction from residual thyrocytes in FNA syringes and technical challenges associated with performing FNA on small-sized nodules may represent confounders affecting the performance metrics of molecular testing (18, 21). In the present study, eight patients were diagnosed with PTC despite testing negative for *BRAF* preoperatively, with subsequent IHC confirming *BRAF* V600E in four cases on FFPE thyroidectomy specimens (Fig. 2). Notably, three of these four patients had PTMC, with tumor sizes less than 10 mm. These findings suggest that false-negative results were likely due to the challenges in aspirating sufficient tumor material rather than assay limitations. Moreover, studies suggest that one of the most frequent challenges in FNA diagnoses is the failure to sample microcarcinomas embedded within an adenomatous goiter, which can lead to diagnostic inaccuracies (22, 23). As nodules harboring microcarcinomas grow larger, precise sampling of the malignant area becomes increasingly difficult, further contributing to diagnostic errors (24, 25).

It is noteworthy that the only one false-positive patient, categorized as TBS III on FNA cytology, was ultimately diagnosed as minimally invasive FTC-staged pT2N0M0 revealing negative VE1 staining. Further Sanger sequencing identified a novel non-V600E *BRAF* mutation, characterized by a duplication of nucleotides from 1794 to 1802, resulting in a protein alteration (V600_K601insNTV), which was first observed in FTC (15). This case highlights the potential benefits of preoperative molecular testing to find out disagreement between preoperative diagnosis and final pathology to uncover novel mutations in thyroid malignancies, contributing to a deeper understanding of oncogenesis.

The strengths of our study include its independence from industry sponsorship, as the entire study was funded by scientific research grants. In addition, the pathologists were blinded to the results of the preoperative molecular testing, ensuring unbiased evaluation. However, several limitations were noted. The sample size was relatively small, and not all pathology specimens underwent IHC VE1 staining as part of routine pathological services. To address this, a best-estimate approach was utilized, incorporating cases with indirect confirmation of mutation status to expand the sample size and enable a more comprehensive statistical analysis. Future research could focus on larger cohorts with routine IHC staining or alternative confirmation methods to validate and extend these findings.

## Conclusion

This prospective study demonstrated that the ThyroSCAN PanelChip effectively identified *BRAF* V600E mutations in thyroid nodules using FNA residual thyrocytes. Future studies incorporating broader panels to detect additional genetic mutations may further enhance the utility to solidify its role in precision diagnosis of thyroid nodules.

## Supplementary materials



## Declaration of interest

There is no conflict of interest that could be perceived as prejudicing the impartiality of the work reported.

## Funding

This work was supported by the National Science and Technology CouncilNational Science and Technology Councilhttps://doi.org/10.13039/100020595, Taiwan (MOST 110-2314-B-075-027-MY3 & NSTC113-2314-B-075-007) and the Taipei Veterans General Hospitalhttps://doi.org/10.13039/501100011912 (V113C-166, V114C-177, V114C-225). These funding agencies had no influence on the study design, data collection or analysis, decision to publish or preparation of the manuscript.
