# Supplementary material for: Validation of preoperative BRAF V600E testing by ThyroSCAN PanelChip in thyroid nodules
Source: Endocr Connect. 2025 May 16;14(5):e240718. doi: 10.1530/EC-24-0718 (PMC12087276; doi:10.1530/EC-24-0718)
Supplement: Supplementary file 1 [file supplementary_materials.pdf]

Supplementary Table 1. Distribution of genetic mutations of all participants across the Bethesda System categories

	TBS I	TBS II	TBS III	TBS IV	TBS V	TBS VI	P value
Total Patients (n)	33 (20.9%)	36 (22.8%)	39 (24.7%)	8 (5.1%)	26 (16.5%)	16 (10.1%)	-
Insufficient genetic material† (n/%)	15 (45.5%)	11 (30.6%)	8 (20.5%)	0 (0.0%)	6 (23.1%)	2 (12.5%)	0.048*
Mutation non-detected‡ (n/%)	16 (48.5%)	20 (55.6%)	24 (61.5%)	5 (62.5%)	10 (38.5%)	2 (12.5%)	0.016*
BRAF V600E Mutation (n/%)	0 (0.0%)	1 (2.8%)	3 (7.7%)	1 (12.5%)	9 (34.6%)	12 (75.0%)	<0.001*
RAS Mutation (n/%)	2 (6.0%)	4 (11.1%)	4 (10.3%)	2 (25.0%)	1 (3.8%)	0 (0.0%)	0.394
NRAS (n/%)	1 (3.0%)	4 (11.1%)	3 (7.7%)	2 (25.0%)	1 (3.8%)	0 (0.0%)	
HRAS (n/%)	1 (3.0%)	0 (0.0%)	1 (2.6%)	0 (0.0%)	0 (0.0%)	0 (0.0%)	
Thyroidectomy (n/%)	6 (18.2%)	5 (13.9%)	17 (43.6%)	6 (75.0%)	24 (92.3%)	15 (93.8%)	<0.001*
ROM (n/%)	3 (50.0%)	2 (40.0%)	5 (29.4%)	2 (33.3%)	20 (83.3%)	15 (100.0%)	<0.001*

* These P values indicate statistical significance.

† Prevent ThyroSCAN from producing meaningful data; ‡ Wild-type gene observed in the specimen; TBS, the Bethesda System, ROM, risk of malignancy
